# Model of the complex of Parathyroid hormone-2 receptor and Tuberoinfundibular peptide of 39 residues

**DOI:** 10.1186/1756-0500-3-270

**Published:** 2010-10-27

**Authors:** Mirna Abraham-Nordling, Bengt Persson, Erik Nordling

**Affiliations:** 1 Division of Surgery, Department of Clinical Sciences, Danderyd Hospital, Karolinska Institutet, Stockholm, Sweden; 2 IFM Bioinformatics, Linköping University, S-581 83 Linköping, Sweden

## Abstract

**Background:**

We aim to propose interactions between the parathyroid hormone-2 receptor (PTH2R) and its ligand the tuberoinfundibular peptide of 39 residues (TIP39) by constructing a homology model of their complex. The two related peptides parathyroid hormone (PTH) and parathyroid hormone related protein (PTHrP) are compared with the complex to examine their interactions.

**Findings:**

In the model, the hydrophobic N-terminus of TIP39 is buried in a hydrophobic part of the central cavity between helices 3 and 7. Comparison of the peptide sequences indicates that the main discriminator between the agonistic peptides TIP39 and PTH and the inactive PTHrP is a tryptophan-phenylalanine replacement. The model indicates that the smaller phenylalanine in PTHrP does not completely occupy the binding site of the larger tryptophan residue in the other peptides. As only TIP39 causes internalisation of the receptor and the primary difference being an aspartic acid in position 7 of TIP39 that interacts with histidine 396 in the receptor, versus isoleucine/histidine residues in the related hormones, this might be a trigger interaction for the events that cause internalisation.

**Conclusions:**

A model is constructed for the complex and a trigger interaction for full agonistic activation between aspartic acid 7 of TIP39 and histidine 396 in the receptor is proposed.

## Findings

### Background

The recent extension of the structural knowledge of both class A and class B G-protein coupled receptors (GPCRs) with the X-ray determination of the human adrenergic β 2 receptor [1], the turkey adrenergic β 1 receptor [2], the human A2A Adenosine receptor [3] and squid rhodopsin [4,5] coupled with the structures of extra-cellular domains (ECDs) of several class B hormone receptors [6-9], including the parathyroid hormone-1 receptor (PTH1R), makes it possible to begin a computational exploration of possible interactions of the parathyroid hormone-2 receptor (PTH2R) [10] and its natural ligand, the tuberoinfundibular peptide of 39 residues (TIP39) [11,12].

The aim of the present study is to propose possible interactions between the hormone and its receptor. There are three related peptide hormones that interact with the two PTH receptors. Parathyroid hormone (PTH) binds and stimulates both receptors resulting in intracellular cAMP release and Ca^2+ ^signalling. TIP39 binds to both receptors, but to PTH2R a hundredfold stronger than to PTH1R and only signal through PTH2R. The parathyroid hormone related protein (PTHrP) binds to and signals only through PTH1R. However, only TIP39 induces β-arrestin and protein kinase Cβ mobilisation and receptor internalisation of PTH2R [13]. A further objective of the study is to investigate how the related peptides can selectively bind to and activate the receptors. The truncated TIP39(7 - 39) is a high affinity antagonist for the PTH1R, while losing much of its affinity for the PTH2R [14], which indicates that the N-terminal portion is involved in both the selectivity between the two receptors and affinity to PTH2R.

The function of the TIP39 - PTH2R system appears to be diverse with primary sites of expression including the nervous system [15], thyroid gland, pancreas, heart, vascular muscle, the reproductive system and lung [16]. An NO-dependent vasodilatory effect of TIP39 was demonstrated if the PTH1R is desensitized by either exogenously administered or endogenously released PTHrP [17,18]. Knockout mice lacking the gene encoding TIP39 are sterile, examination of the testes shows that they contain no spermatids. By antibody labelling of the chromosome spreads it was shown that spermatogonia do not complete the prophase of meiosis I [19]. They also display a more stress anxiety prone phenotype in behaviour tests than wild type mice [20]. Another study has shown that the TIP39 - PTH2R system is activated in response to acoustic stress [21]. A recent report also points out a connection to neuropathic pain, where PTH2R was shown to be selectively localized on myelinated A-fibers. Pharmacological studies showed that TIP39 induced nociceptive responses that were mediated by activation of G_s _and cAMP-dependent protein kinase. It was found that nociceptive responses induced by TIP39 were significantly greater following partial sciatic nerve injury induced neuropathic pain, without changes in PTH2R expression [22].

There is a possibility of using TIP39 as a tool to investigate the function of PTH2R in more detail by defining its interaction sites with PTH2R and it may also be possible to use it as a source of antagonistic peptides as the truncated TIP39(7 - 39) is for the PTH1R. That possibility could be of interest to modulate the pain sensation in neuropathic pain.

A class B GPCR of subfamily B1, a hormone receptor, has an extracellular N-terminal domain (ECD), a transmembrane (TM) domain and an intracellular C-terminal domain [23]. The fold of the TM region was revealed when the first structure of a GPCR was crystallographically determined (bovine rhodopsin [24]) and it consists of seven α-helices that form an anti-parallel seven-member helical bundle. As the ECD of PTH1R has been crystallized in complex with PTH it is possible to construct a model which captures both the binding of the C-terminal part of the hormone to the ECD and its interactions with the transmembrane region of the receptor. As the current structure of the TM region of GPCRs is from class A and only distantly related to class B, it is reassuring that biophysical studies indicate that the eighth helix of the class A GPCRs, which is positioned parallel to the membrane, also is present among the class B GPCRs. However, no apparent sequence homology is detected in that part of the receptor [25]. In contrast to the available class A structures, PTH2R has a large extracellular loop 1 (ECL1) between TM helix 2 and 3, which consist of 31 amino acid residues. In the case of ECL1, NMR experiments of the corresponding part of PTH1R have indicated that it should form a central helix comprising residues 256 - 264, which is shown to contact the hormone with a centrally placed Leu261 binding to Lys27 of the hormone [26]. As no structure has been publically made available, the loop has been left to the prediction scheme with the added notion that the prediction accuracy might be low for this region.

As the intracellular part of the receptor lacks a homologue with known structure we do not attempt to incorporate that domain in the model.

NMR experiments of TIP39 indicate that it has two helical regions comprising the residues 5 - 19 and 27 - 34, and that the two helical regions are tilted in relation to each other [27]. Similar experiments of PTH fragments 1 - 34 and 1 - 39 also indicate two helical regions connected by a loop region consisting of His14 to Ser17 [28]. However, X-ray studies of PTH(1 - 34) show a single slightly bent helical region comprising residues 3 - 33, with the bend located between residues 12 and 21 [29]. This is in accordance with previous studies of the bioactive conformation of PTH, where lactam bridges were used to trap the hormone in rigid helical conformations and an increase of the activity was seen at conformations that were in compliance with the extended helical model of the hormone [30]. The extended conformation of PTH was also used to construct a model of PTH's interaction with the PTH1R TM region, where prominent interactions involve Ser1 and Lys13 of PTH and Met425 and Arg186 of the receptor [29]. As the ECD structures for class B GPCRs were not available at the time of the modelling, the authors could not propose a model of that domain. NMR structures of PTHrP(1 - 36) also show two helical regions, His5 - Leu8 and Gln16 - Leu27, connected by a loop region. Analysis of the 30 conformations deposited in the protein databank entry 1BZG [31] shows that 12 of the conformations have an irregular helical conformation in the loop region which leads us to the conclusion that the extended conformation shown to be bioactive for PTH might also be active for TIP39 and PTHrP.

## Methods

The sequences of TIP39, PTH2R and PTHrP were retrieved from the Uniprot protein sequence database with identifiers TIP39_HUMAN [Swiss-Prot:Q96A98], PTH2R_HUMAN [Swiss-Prot:P49190] and PTHR_HUMAN [Swiss-Prot:P12272] [32]. The first 24 amino acid **residues **of PTH2R that form the signal peptide were removed to yield the mature chain of PTH2R. The numbering of the PTH2R model is based on the full sequence of the Uniprot entry PTH2R_HUMAN [Swiss-Prot:P12272], starting with 1 at the first residue of the signal peptide.

For the extracellular domain of PTH2R and TIP39, the complex between PTH1R and PTH was used as template [PDB:3C4M] [7]. The alignment of the ECD domains was generated using MUSCLE [33].

The evaluation of the most appropriate template for the TM region is performed by the method presented in Worth *et al*. 2009 [34], which is based on the existence of certain key structural features in each TM helix and sequence similarity where multiple receptors has the same structural feature. They also performed a structural alignment of the available GPCR X-ray structures which we extracted from their paper for use in the evaluation process. The subfamily B1 of the human class B GPCRs was aligned based on the alignment presented in Harmar 2001 [23]. As profile - profile alignments are shown to be more sensitive for transmembrane proteins with decent alignment accuracy for proteins with sequence identities of between 10 and 20% [35], a profile - profile alignment of the two alignments was constructed using MUSCLE [33]. By following the published scheme the following templates are suitable for the different transmembrane helices, TM1: 2VT4 [PDB: 2VT4], TM2: 2RH1 [PDB: 2RH1], TM3: 2VT4 [PDB: 2VT4] or 2RH1 [PDB: 2RH1], TM4: 2VT4 [PDB: 2VT4], TM5: 1U19 [PDB: 1U19], TM6: 2VT4 [PDB: 2VT4], TM7: 1U19 [PDB: 1U19]. As 2VT4 [PDB: 2VT4] was the highest scoring template for four of the seven helices it was selected as the most suitable template to base a model upon.

The resulting alignment to the most similar template was subsequently manually adjusted in the loop regions to conserve disulfide bridges of the overall GPCR fold. A crude model was built to inspect how the structure was affected by insertions and deletions in the alignment. In cases were an insertion caused the amino acid chain to create a bulge before continuing in the track of the template, was the insertion extended in order to sample a larger portion of the loop to find a low energy conformation. For deletions, the major concern is that the backbone will assume a strained conformation, thus it may require that a few more residues are left unaligned at the edges of the deleted region. This procedure is required due to the nature of the modelling procedure where the software aims to preserve structural elements derived from the template. Based on these operations the resulting alignment deviates from the evolutionary alignment for regions that have evolved to contain different structural characteristics than in the template. This is evident by the artificially created gaps at positions 32-37, 144-145, 179-180, 244-245, 276-277, 283-285, 322-323, 364-365, 390-392 to allow the modelling software to perform an efficient sampling of loop conformations to find a low energy conformation for those regions.

The software used for the homology modelling was ICM (Molsoft LLC, San Diego, CA, USA) [36]. The modelling was performed using the Bioinfo module. The procedure includes side-chain optimisation and loop sampling performed according to the ICM scripts.

The structures of TIP39 and PTHrP(1 - 36) were modelled on the X-ray structure of PTH(1 - 34) in 1ET1 [PDB:1ET1] [29], which subsequently were superimposed on the C-terminal part of PTH in the complex of 3C4M [PDB:3C4M] [7]. Even though the sequence identity is limited to 15%, common themes are evident in the three related hormones TIP39, PTH and PTHrP. The alignment was generated using MUSCLE [33].

The model of the PTH2R - TIP39 complex was constructed by placing the ECD model in such a manner that TIP39 could reach into the transmembrane region and interact with the top of TM3 and TM7, a region which has been experimentally verified to discriminate binding of PTHrP to PTH2R [37,38]. The sequence of the ECD and the TM region of the PTH2R chain was threaded through the placed parts and a combined model was constructed. The construction of the model in complex with TIP39 required an additional step of refinement of ECL1 due to sterical clashes with the hormone. Using the loop sampling feature of ICM, the third highest ranking conformation was selected as it was the most frequently visited conformation during the sampling. The region between the ECD and the TM region was also refined using loop sampling. The most frequently visited conformation which also was the lowest energy conformation was selected. Unfavourable interactions in the complex were relieved by iterative minimizations using restraints placed on the backbone atoms of the TM region of the receptor.

The structural model of the TIP39 - PTH2R complex is available in the Protein Model DataBase [39,40] under the accession number PM0076250.

Complexes with the hormones PTH and PTHrP and PTH2R were built by superimposing them upon the TIP39 - PTH2R complex, followed by energy minimisation.

## Results

The scheme used to select template for the TM region resulted in that 2VT4 [PDB:2VT4] was found to be the most appropriate template, with chain b showing electron density for the largest portion of the residues and 2VT4 was therefore used. The overall sequence identity to 2VT4 was 11%, the highest of available GPCRs with known structure. The TM regions showed identities ranging from 7 to 19%.

**Figure 1:**
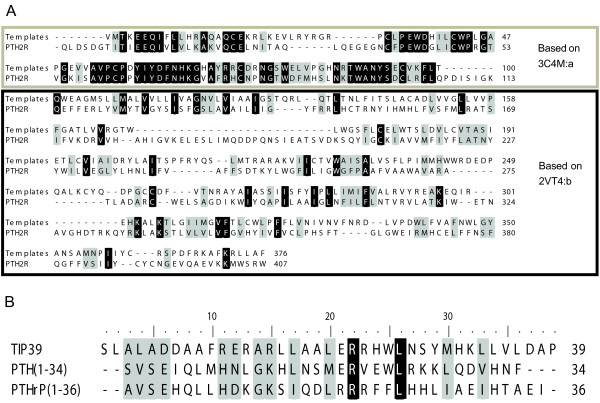
**Alignments**. Black shaded residues are identical and grey shaded residues are similar according to PAM250 similarity matrix. **A: **Alignment between PTH2R and the templates used for homology modelling; thick border box - extracellular domain; thin border box - transmembrane region; no box - intracellular domain. **B: **Alignment of tuberoinfundibular peptide of 39 residues (TIP39), parathyroid hormone (PTH) and parathyroid hormone related protein (PTHrP). The amphipatic nature of the peptides is visible through the pattern of conserved hydrophobic residues in the C-terminal portion of the alignment.

Figure 1A shows the alignment of PTH2R and the templates used in the modelling. The ECD shows 50% residue identity while the TM region shows only 11%. Seven residues form the linker region between the ECD and the TM region. It is noticeable that ECL1 (residues 179 - 205 in the mature chain of PTH2R) is longer than in the template and thereby lacks a structural template. ECL2 (residues 276 - 285) which contains the conserved disulfide in the GPCR super-family is shorter than in β1R, which contain a helical region.

The alignment of the hormones is shown in Figure 1B, and even though the sequence identity is only 15%, several common themes are evident in the three related hormones TIP39, PTH and PTHrP. The amphipatic nature of the peptides can be traced through the conserved pattern of hydrophobic residues in the C-terminal portion of the alignment. Also worth noticing is the conserved basic residues at positions 15 and 22 (using TIP39 numbering).

**Figure 2:**
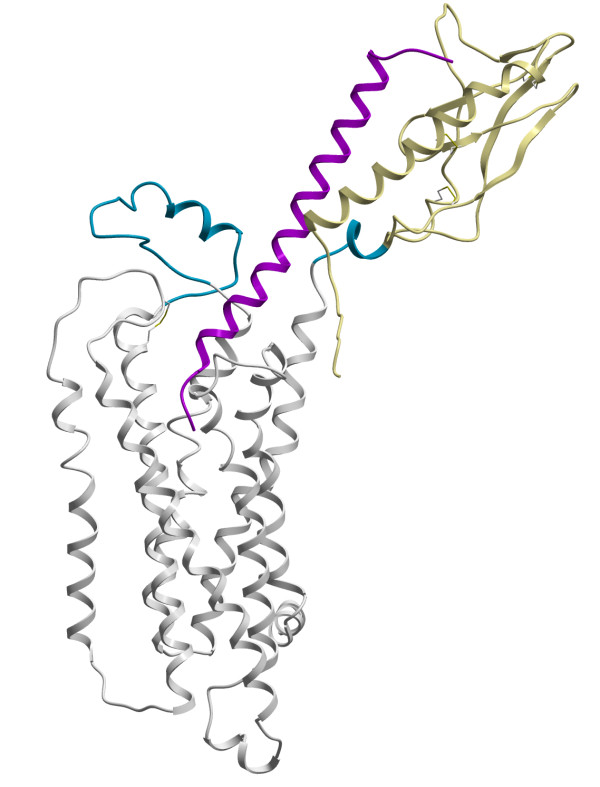
**Structure of predicted complex**. The PTH2R model in ribbon representation, regions without template in blue, the extracellular domain in khaki and the TM region in white and the tuberoinfundibular peptide of 39 residues in magenta. TM helix 1 is located to the right and 4 to the far left.

The structure follows the general fold of the GPCR family (see Figure 2) with the ECD positioned (coloured khaki) above TM helix 1, directing the N-terminus of the hormone into the central cavity of the TM helical bundle (coloured white), making contact with the extracellular ends of TM helices 3 and 7. The regions without template, the seven residues between the ECD and the TM region and the large ECL1 are coloured blue. The loop sampling of the ECL1 indicated an unordered structure with a central helical region flanked by two loops connecting TM helix 2 and 3. A disulfide bond is formed between Cys236 in the top of TM3 and Cys306 in ECL2. As ECL2 is shorter than in the template no helix can be formed and the loop has an extended conformation.

**Figure 3:**
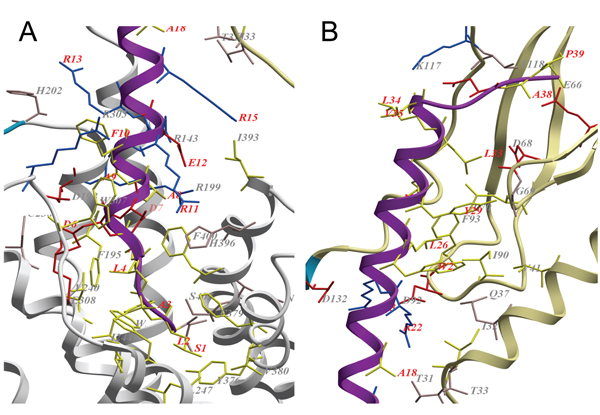
**Close-up of the binding region between PTH2R and the tuberoinfundibular peptide of 39 residues**. The extracellular domain (ECD) in khaki, regions without template in blue, the TM region in white and the tuberoinfundibular peptide of 39 residues (TIP39) in magenta. Residues in the binding interface of TIP39 and the receptor (within 3Å distance of each other) are shown in sticks. Acidic residues are coloured red, polar residues are coloured pink, basic residues are coloured blue and hydrophobic residues are coloured yellow. Residues in TIP39 are labelled in red and residues in the receptor are labelled in gray. **A**. Interactions of the N-terminus of TIP39 with the TM region. **B. **Interactions of the C-terminus of TIP39 with the ECD.

The interactions with TIP39 and the receptor are partly defined by polar interactions of basic, acidic and polar residues (shown in blue, red and pink, respectively, in Figure 3), and partly defined by hydrophobic interactions (marked with yellow sticks in Figure 3).

**Figure 4:**
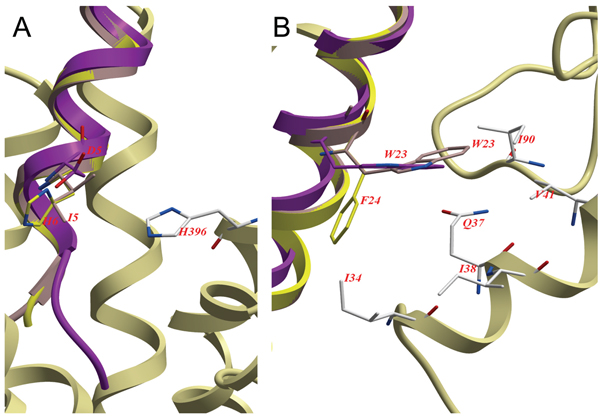
**Interactions between PTH2R and the investigated hormones**. Tuberoinfundibular peptide of 39 residues in magenta, parathyroid hormone in pink and parathyroid related protein in yellow. **A**. The central interaction of Asp7, Ile5, His5 with His396 of TM helix 7 of the receptor. **B**. Trp25, 23 and Phe23 is shown with interacting residues Ile34, Gln37, Ile38, Val41 and Ile90 in the ECD of the receptor.

Primary differences between the hormones TIP39 and PTH that bind and at least partially activate PTH2R and PTHrP which lacks binding affinity, include Phe23 in PTHrP which corresponds to a Trp in the agonistic peptides, interacting with a mainly hydrophobic environment consisting of residues Ile10, Gln13, Ile14, Val17 and Ile66 in the ECD (see Figure 4B), and His5 corresponding to Asp in TIP39 and Leu in PTH and which is placed in the vicinity of His396 of TM helix 7 (see Figure 4A).

## Discussion

We have calculated a model of PTH2R based upon template structures for the ECD and the TM region. The most reliable region of the model is the ECD, as it is modelled on the related PTH1R. A sequence identity of 50% can reliably give a model of good resolution. The major difference in the alignment of the two parathyroid receptors ECDs originates from that the shorter loop of PTH2R of residue 32 - 37 is unaligned. This allows the modelling software to find a low energy conformation that connects the conserved structure elements prior to, and after the loop. The low sequence identity within the TM region is of course a challenge for the construction of a reliable model. Previous studies of divergent protein families with a common fold show that models can be created for proteins with sequence identity as low as 20% [41]. Within the GPCR super-family, bovine rhodopsin has 18% sequence identity to the human adrenergic β 2 receptor, while still sharing the same fold with an all-atom RMSD of 3 Å for the TM region [34]. In Vohra *et al*. 2007 [42], a plant GPCR named GCR1 was used to construct an alignment of the TM region of GPCR class A and B, as it had sequence similarity to both classes. Their published alignment is in accordance with our alignment of the TM regions [42], indicating that the alignment of the TM helices may indeed be correct.

An additional issue with the model is the large ECL1. The loop sampling of ICM can not reliably predict such large loops, although structural characterisation of ECL1 of the PTH1R has shown the existence of a central helical region of the loop that interact with PTH, hinting at that some of the loops characteristics are found by the modelling process [26].

**Figure 5:**
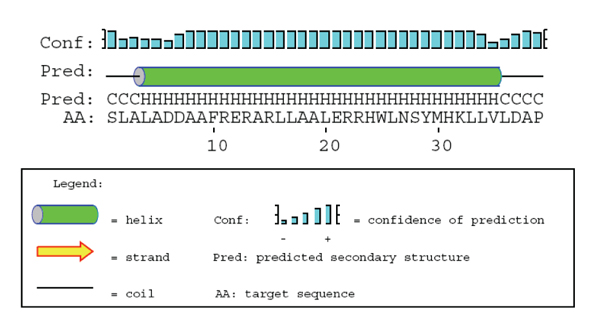
**Secondary structure prediction for TIP39**. Prediction of the secondary structure of tuberoinfundibular peptide of 39 residues by PSIPRED.

The conformation of TIP39 is likely to be predicted with a fair amount of precision. The template is the parathyroid hormone, where both X-ray [29] and constrained helical analogues, which are forced to adopt a continuous helical conformation, indicate that a slightly twisted single helix is the bioactive conformation of PTH [30]. A previous study of the TIP39 structure using a combined approach based on NMR and molecular dynamics indicates the presence of two helical regions in TIP39, residues 5 - 19 and 27 - 34 with a flexible linker joining them [27]. Similar findings are evident in NMR structures of PTH [28]. However, examining the ensemble of structures generated in the experiments one can find examples of conformations that display a single elongated helical region. In Jin *et al*. 2000, the authors speculate that the discrepancy between the solution structure and their X-ray structure involves the fact that PTH is a hydrophobic peptide which might need a more hydrophobic environment to adopt its bioactive conformation [29]. A similar situation could be envisioned for TIP39. Another indication that a single helical conformation is plausible is that a PSIPRED secondary structure prediction for TIP39 indicates that it is a single helical structure ranging from residue 4 - 35 (Figure 5) [43].

The model of the complex places the N-terminus of the hormone in the central cavity of the TM helical bundle, allowing its residues to be positioned in the TM region and interact directly with the receptor. The importance of the N-terminal part has been highlighted by the fact that deletion of the six first residues makes the remainder a potent antagonist of the PTH1R, while losing much of the strength of the binding to the PTH2R [14,44]. In Figure 3A, one can see that the first residues of TIP39 bind in a hydrophobic site in the central cavity. The only polar residue in the N-terminus, Ser1 in TIP39 are in position to form a hydrogen bond with Ser403 in TM7. Positions in PTH2R which have been shown to be directly involved in the discrimination between the PTH and PTHrP for PTH2R; Ile244 (interacts with Leu2 and Ala3 in TIP39 in the model of the complex) in TM3 and Cys397 (matched to Glu12 in TIP39) and Phe400 (in proximity of Ala5 and Ala8 in TIP39) in TM7 are in close contact with the placed ligand [38]. The major mismatch of the TIP39 residues in the central cavity are Asp6 which is positioned into a cavity between TM helix 3 and 4, its closest match is Asp198 in an otherwise hydrophobic environment. His396 in TM7 are a more suitable interaction partner for both Asp6 and Asp7, which could be accomplished by a slight rotation of the hormone. A further interaction is the salt bridge formed by Glu12 of TIP39 and Arg143 and Arg199 of the receptor.

The interaction of the hormone with the ECD (see Figure 3B) is characterised by hydrophobic forces of Trp25, Leu26, Tyr29, Met30 and Leu 33 that interact with a continuous surface made up by Val41, Leu70, Ile90 and Phe93 in the ECD. These interactions are supported by those of Arg22 and Arg23 in TIP39 with Asp92 and Asp132 in the receptor.

The modelling of the complexes of related peptides hormones, TIP39, PTH and PTHrP with PTH2R show that the mutationally verified importance of the Trp at position 25 in TIP39 and at position 23 in PTH in binding to the receptor, while the smaller Phe in PTHrP can not fill the binding site (see Figure 1B and 4B). Further it can be noted that His5 of PTHrP is placed in contact with His396 of the receptor, mutation of that position to Ile, the corresponding residue of PTH, restores activity in combination of mutation of Phe23 to Trp [38]. Lingering on the interaction with His396, TIP39 has a Asp at the corresponding site and as it causes the receptor to internalize and activates further signalling pathways than PTH [13], it is tempting to speculate that TIP39s possibility to evoke those signalling responses lies in that acidic residue, unique for it among the related hormones (see Figure 1B and 4A). The hypothesis could be tested by the mutation of the Ile5 of PTH to Asp in order to explore if that mutant can fully activate PTH2R. Of the 43 reported orthologues in the ENSEMBL database to PTH2R, all except the one from the nine-banded armadillo (*Dasypus novemcinctus*) have a conserved His at that position, while the armadillo has an Arg which still could interact with an acidic residue. Unfortunately, there is no reported TIP39 sequence for *Dasypus novemcinctus*, but all 23 reported TIP39 orthologues have two aspartic acids at positions 6 and 7 [45].

## Conclusions

In this work, we present a hypothesis of how TIP39 could bind to PTH2R and how the receptor may discriminate among the related peptides TIP39, PTH and PTHrP. Furthermore, we propose that the peptide activates the receptor in a single helical conformation as seems to be the case for PTH. We also propose a mutant of PTH, Ile5Asp, which we speculate could act as a full agonist and cause internalisation of the receptor. We hope that this study may provide insight into an interesting system that deserves more attention as its full implications are just beginning to be unravelled. The connection to neuropathic pain suggests a new route to modulate the pain sensation via the TIP39 - PTH2R system, which today is an area of great unmet medical need.

## Competing interests

Erik Nordling is, besides being affiliated with IFM Bioinformatics, Linköping University, also employed by Biovitrum AB, which has no interest in the current article. The other authors declare that they have no competing interests.

## Authors' contributions

MAN participated in the design of the study, sequence alignment and draft of the manuscript. BP participated in the design of the study and revision of the manuscript. EN participated in the design of the study, model building and draft of the manuscript. All authors read and approved the final manuscript.
